# Assessment of Trace Elements Supply in Canned Tuna Fish Commercialized for Human Consumption in Brazil

**DOI:** 10.3390/ijerph182212002

**Published:** 2021-11-16

**Authors:** Nayara Vieira de Lima, Daniela Granja Arakaki, Elaine Silva de Pádua Melo, David Johane Machate, Valter Aragão do Nascimento

**Affiliations:** 1 Group of Spectroscopy and Bioinformatics Applied Biodiversity and Health (GEBABS), Graduate Program in Health and Development in the Central-West Region of Brazil, Federal University of Mato Grosso do Sul, Campo Grande 79079-900, Brazil; nayaralima.01@hotmail.com (N.V.d.L.); daniarakaki@gmail.com (D.G.A.); elainespmelo@hotmail.com (E.S.d.P.M.); machatedavidjohanemachate@yahoo.com.br (D.J.M.); 2 Graduate Program in Sciences of Materials, Federal University of Mato Grosso do Sul, Campo Grande 79079-900, Brazil

**Keywords:** metalloid, (non)metal, minerals, macro- and microelements, health risk, pollutant, processed fish

## Abstract

This study evaluates the elemental content in 4 types of canned tuna fish groups, each with 4 brands that are commercialized for human consumption in Brazil. The results are based on trace elements in canned tuna fish quantified by ICP OES and a comparison to limit levels set by the FAO/WHO. We also checked the carcinogenic risk (CR), non-carcinogenic risk (Hazard Index (HI) and Hazard Quotient (HQ)), and pollution index (PI) for the studied canned tuna samples. As and Se concentrations in all groups are above the intake values set by FAO/WHO considering specific groups. The carcinogenic risk values for arsenic (As) in groups are considerably unacceptable (≥10^−4^). Hazard quotients (HQ) were >1 for As in all groups, while no sample was below 1 for HI. The pollution index (PI) results show that the main canned tuna fish contaminant is aluminum, then selenium and arsenic, respectively. Only half of the samples did not present elemental contaminant levels. All studied brands of canned tuna presented elemental concentrations that could pose a health risk to human consumption, that could be from CR, HQ, HI, or PI. The contaminant levels are alarming and should raise a red flag for the intake of these products, especially a long-term one. These results urge the authorities to supervise and enforce better practices for this type of food, protecting their population from health hazards.

## 1. Introduction

Worldwide increasing natural resources usage, including land, leads to the spread of several heavy metals and metalloids from modern agriculture processes and motorized vehicle pollution [1]. The contamination of the soil, water, and river basin is highly associated with the massive food production in agriculture and the increased industry process of pesticides and fertilizers production and use; plus, residential source sewage/sludge pollution, intensive mining industries, and natural sources of heavy metals in soil and food crops for animal and human intake [2,3,4,5].

Wind and water flow carry several chemical elements to lakes, streams, and rivers during drought and rain season, including heavy metals, metals, and metalloids [6]. These watercourses and winds are directly linked to seas and oceans metals and metalloids accumulation, becoming pollutants and contaminant matter to plankton and animals, and fishes used for human food [7].

Fish consumption is recommended worldwide, as fish are a source of macro- and micro-elements, vitamins B12 and D [8], protein [9], and omega-3 polyunsaturated fatty acids (eicosapentaenoic and docosahexaenoic acids), which increase the health benefits for humans, lowering obesity, weight gain, body mass index, insulin resistance, type 2 diabetes mellitus, inflammatory bowel diseases, and maintain harmony gut microbiota balance [10]. The mentioned benefits boosted worldwide fish consumption to 21 kg/person/year [11].

Although the canning procedure is suitable for preserving food, this does not mean that such food is not subject to chemical elements contamination. Some countries have conducted several studies on canned fish commercialized in local markets, quantifying and monitoring heavy metals and metalloids concentration, and guaranteeing food safety and public health for consumers [12,13,14,15,16,17,18,19,20,21,22,23]. Studies have shown that heavy metals, metals, and metalloids in some canned fish samples [12,13,14,15,16,17,18,20,21,22,23,24] are a factor of concern; some chemical elements are toxic and can harm health. Arsenic exposure is associated with liver, lung, prostate, and bladder cancers [25]. Other chemical elements such as Al are toxic [26] and are related to diseases such as autism spectrum disorder and multiple sclerosis [27]. Zinc plays a role in numerous biochemical processes in humans and animals; however, overexposure to zinc is related to toxic effects [28]. Excessive iron intake can lead to free radicals linked to oxidative stress, mood disorders, and other diseases [29]. Selenium is nutritionally essential for humans, while it becomes toxic in high doses [30]. An excess of barium can occur, provoking kidney diseases, neurological impairments, cardiovascular, mental, and metabolic disorders [31]. The presence of trace metals in marine waters may happen naturally (from the erosion course of rocks and spoils) [32], or by human activities, from the industrialization and urbanization process [33].

Thus, elemental content surveillance of canned fish is necessary, and should be carried out periodically in several countries.

Based on data published by Lima et al. [24], the purpose of this study is to conduct a human health risk assessment due to the ingestion of chemical elements such as aluminum (Al), arsenic (As), barium (Ba), calcium (Ca), copper (Cu), iron (Fe), selenium (Se) and zinc (Zn) in 4 types of canned tuna fish: (i) natural grated tuna (NGT), (ii) oil grated tuna (OGT), (iii) solid natural tuna (SNT), and (iv) solid tuna in oil (STO). There are four brands of the company that sell these 4 types of condiments in canned tuna in Brazil.

The concentration of these trace elements was compared with the reference values for daily intake of trace elements [34,35,36,37,38,39].

The study showed that carcinogenic risk values for As are above acceptable values. In addition, Al, Se, and As are the principal pollutants with samples achieving pollution index (PI) values above 1.

## 2. Materials and Methods

### 2.1. Tuna Fish Samples Acquisition and Preparation

A total of 48 samples of canned tuna fish of different brands were purchased in supermarkets in Campo Grande, Mato Grosso do Sul, Brazil. In this study, two main types of canned tuna fish were considered: grated and solid. Each of the four brands (G, C, O, and P) sells 4 types of canned tuna fish: (i) natural grated tuna (NGT), (ii) oil grated tuna (OGT), (iii) solid natural tuna (SNT), and (iv) solid oil tuna (STO). The natural samples are respective to those in brine rather than oil. A detailed description of analyzed brands of companies and types of canned tuna fish is presented in the work of Lima et al. (2021) [24].

### 2.2. Microwave-Assisted Digestion Procedure, Inductively Coupled Plasma–Optical Emission Spectrometry (ICP OES) Elemental Analysis, and Calibration Curves

Procedures were taken as described by Lima et al. [24]. About 400 mg of the canned tuna samples of each type and from different companies were accurately weighed in a Teflon digestion vessel. Next, 1 mL of nitric acid and 3 mL of hydrogen peroxide were added. Digestion of samples was carried out in a microwave digestion system. All the digestion analyses steps were conducted in triplicate.

The procedure for quantifying Al, As, Ba, Ca, Cu, Fe, Se, and Zn in canned tuna using ICP OES, analytical calibration curve, the limit of detection (LOD), limit of quantification (LOQ), and correlation coefficient (R^2^) are described by Lima et al. (2021) [24]. The LOD was calculated as three times the standard deviation of the blank signal (B) expressed in concentration divided by the slope of the analytical curve (AC): LOD = 3*B/AC, and the LOQ was obtained as ten times the standard deviation of the blank divided by the slope of the analytical curve: LOQ = 10*B/AC [24,40,41].

An addition/recovery test for the elements under study was conducted in a tuna fish sample by spiking 0.5 mg/L of each analyte. The method had a recovery interval of 80–110% for the spike 0.5 mg/L, which is between 80–120% of the previously established limit proposed by the Union of Pure and Applied Chemistry (IUPAC) and Association of Official Analytical Chemists (AOAC) [40,42,43,44].

### 2.3. Human Health Risk Assessment and Pollution Index

Carcinogenic risk estimates represent the probability that an individual will develop cancer over a lifetime due to a specific exposure to a carcinogenic chemical; that is, exposure to daily doses over the years of life. Carcinogenic risk (CR) is calculated by the following equation:

(1) Carcinogenic Risk=CDI ×SF 

CDI is the chronic daily intake dose of carcinogenic elements (mg/kg/day), and carcinogenic risk (CR) is quantified by the chemical element cancer slope factor (SF). The SF results from the application of a low-dose extrapolation procedure, presented as “mg/kg/day” [45]. Their units are the inverse of the lifetime average daily dose CDI, because the ratio is a probability (i.e., unitless). The SF of As is 1.5/mg/kg/day. The cancer risk is a sum of individual carcinogenic elements in different exposure pathways within total cancer (R). According to the United States Environmental Protection Agency (US EPA) [46], the value of acceptable cancer risk ranges from 10^−6^ to 10^−4^, while values >10^−4^ are considered unacceptable.

The human health risk of heavy metal intake was evaluated based on the chronic daily intake dose (CDI) for a chemical contaminant in the tuna fish over the exposure period and the fish intake quantity. CDI (mg/kg/day) was calculated using the following Equation (2):

(2) $$\mathrm{CDI}=\frac{C_{\mathrm{tuna}\;\mathrm{fish}}\times {\mathrm{IR}}_{\mathrm{tuna}\;\mathrm{fish}}\times \mathrm{EF}\;\times \mathrm{ED}}{\mathrm{BW}\;\times \mathrm{AT}}\;$$

where CDI is the chronic daily tuna fish intake dose; C_tuna fish_ is the concentration of the chemical elements present in samples (mg/kg) sold by companies (G, C, O, and P) in different types of canned fish (NGT, OGT, SNT, and STO) [24]; IR_tuna fish_ is the ingestion rate (130 g/day); EF is the exposure frequency (3 times per week = 156 days/year) as recommended by FDA and EPA [42,45]; ED is the exposure duration (life exposure = 8, 18 and 30 years); BW is the body weight (kg), and we considered 26 kg for an 8-year-old; 62 kg for an 18-year-old, and 70 kg for a 30-year-old. The AT is the average time (AT = ED × 365 days/year). The average daily fish consumption was set as 130 g/day, which is near the recommended amount [47,48], and it is the portion of choice once it is the content of one canned tuna.

The non-carcinogenic health risk to humans by the intake of heavy metal-contaminated fish was obtained using a hazard quotient (HQ), which is a ratio of CDI and chronic oral reference dose (RfD), determined by the following Equation (3):

(3) $$\mathrm{HQ}=\frac{\mathrm{CDI}}{\mathrm{RfD}}$$

The RfD values for the risk calculation were established by the Joint Food and Agriculture Organization/World Health Organization (FAO/WHO) Expert Committee on Food Additives “Food safety and quality: Summary reports”, [37] and the regional screening levels for use by risk assessors in site screening for chemical contaminants for the assessment of human health, which the RfD values for the elements were established: Al = 1.0 mg/kg/day; As = 0.0003 mg/kg/day; Ba = 0.2 mg/kg/day; Ca = not available; Cu = 0.04 mg/kg/day; Fe = 0.7 mg/kg/day; Se = 0.005 mg/kg/day; Zn = 0.3 mg/kg/day [49,50]. As show in Equation (3), a toxic risk is considered to occur if HQ > 1, whereas HQ < 1 represents a negligible hazard (adverse non-carcinogenic effects) [51].

Another critical concept related to the HQ is the HI. It is the sum of the risk quotients for simultaneous exposure to two or more metals; that is, HI = HQ_Al_ + HQ_As_ + HQ_Ba_ + HQ_Cu_ + HQ_Fe_ + HQ_Se_. If HI < 1, canned tuna fish consumption is safe, while in the case of HI > 1, canned tuna fish consumption may pose a health risk [51].

The pollution index (PI) was calculated following Equation (4) adapted by Adebiyi et al. [47]. For PI > 1 values, there is an assumption of contaminated samples, whereas PI < 1 stand for non-contaminated samples.

(4) $$\mathrm{PI}=\frac{\mathrm{Cn}}{\mathrm{AC}}\;$$

where Cn—chemical element concentration in tuna fish and AC—acceptable values limit in food: Al = 1 μg/g [52]; Fe = 43 μg/g in food [53]; Zn = 50 µg/g [53]; As = 2 μg/g [54]; Cu = 40 µg/g [53], and Pb = 0.4 μg/g [55]. For Se, we adopted the Tolerable Upper Intake Level (UL), since there is currently no permissible limit for the element in fish.

### 2.4. Statistical Analysis

The data were analyzed by two-way ANOVA using the GraphPad Prism 8 software version 8.0 for Windows (GraphPad Software, San Diego, CA, USA). The considered sources of variations were sample brands, and structure (grated or solid), and solvent. The significance of the differences between the means for the individual trace element was considered at *p* < 0.05.

## 3. Results

The results for all canned tuna fish were represented by two sub-sections. Section 3.1 presents data on the concentration of the trace elements quantified in canned tuna fish purchased in Brazil. Section 3.2 describes the results of the CR, HQ, HI, and PI of the trace elements, based on ingestion of 130 g/day of canned tuna fish for individuals aged 8, 18, and 30 years old.

### 3.1. Canned Tuna Content and Intake Limits

In this study, the concentrations of Al, As, Ba, Ca, Cu, Fe, Se, and Zn in NGT, OGT, SNT, and STO samples in units of mg/kg were converted to mg per 130 g, once this is the canned tuna net weight sold in Brazilian markets. Table 1 presents the elemental concentration for each type of canned tuna and its brand. Al, Ba, Ca, Se, and Cu contents in some canned foods are below the detection limit (<LOD), while Cd and Pb were below the LOD for all samples.

The level of Al in NGT, SNT, and STO groups ranged from 0.00065 ± 1.3 × 10^−5^ to 6.1 ± 0.4 mg/130 g (Table 1). The structure and solvent (how the tuna is presented in the can: either solid or grated; and in which solvent it is stored: either brine or oil) contributed 79.08% to the total aluminum variation among samples (*p* < 0.0001).

The arsenic (As) concentration variations in NGT, OGT, SNT, and STO groups were between 0.2 ± 0.003 and 0.3 ± 0.01 mg/130 g (Table 1), and did not statistically differ among samples.

**Figure 1:**
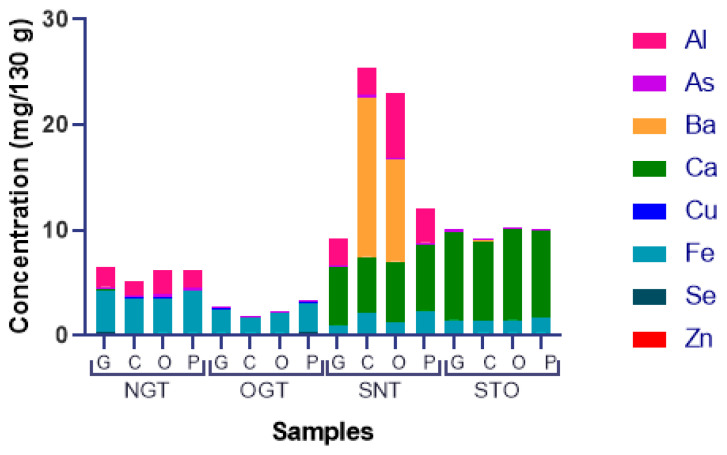
Elemental content distribution in canned tuna fish quantified by ICP OES. NGT = Natural Grated Tuna; OGT = Oil Grated Tuna; SNT = Solid Natural Tuna; STO = Solid Tuna in Oil.

Barium concentrations in canned tuna were between 0.002 ± 0.0003 and 15 ± 4 mg/130 g (Table 1). Barium showed high concentrations only in samples of natural solid tuna in two brands (Figure 1). The structure and solvent contributed to 34.88% of the variation (*p* = 0.0001), while the brand of the canned tuna accounted for 12.82% (*p* = 0.0131). The interaction between these factors represented 38.38% of the variation in barium content (*p* = 0.0029).

Calcium contents ranged from 0.01 ± 0.003 to 9.2 ± 2.6 mg/130 g (Table 1). Structure and solvent were the main sources (95.79%; *p* < 0.0001) of calcium variations in samples, with high calcium content in solid samples only.

Copper concentrations in samples varied from 0.008 ± 0.002 to 0.1 ± 0.01 mg/130 g (Table 1). Solvent and structure were critical to copper variation in samples, accounting for 61.4% (*p* < 0.0001), followed by the interaction with the brands (28.74%; *p* = 0.0001); grated samples and samples in oil registered higher copper amounts. The brand alone was responsible for only 3.88% of variations (*p* = 0.04).

Iron concentrations in canned tuna samples were between 1 ± 0.06 and 4 ± 0.2 mg/130 g (Table 1). The two-way ANOVA identified that variations in iron were due to structure and solvent (81.94%; *p* < 0.0001), brand (8.045%; *p* < 0.0001), and the interaction between these factors (7.525%, *p* = 0.018).

The concentration of Se in canned tuna samples ranged from 0.2 ± 0.005 to 0.3 ± 0.02 mg/130 g (Table 1). The variations in the amount of selenium mainly depend on the structure and solvent (53.01%; *p* < 0.0001), while the brand represents just 8.064% of the difference, and the interaction between factors is 37.17%.

Zinc (Zn) concentrations varied between 0.01 and 0.05 mg/130 g (Table 1). Zinc quantities varied according to structure and solvent (52.04%, *p* < 0.0001), followed by the interaction between factors (33.70%, *p* < 0.0001) and brand (13.43%, *p* < 0.0001).

Elements that are not essential displayed a considerable detectable amount in the samples (Figure 1).

The natural samples (brine—NGT and SNT), aluminum detection presented a substantial amount opposite to those in oil. In the same way, all samples showed the presence of arsenic, which is not only not essential, but also toxic.

### 3.2. Health Risk Assessment

Table 2 shows the CR calculated using Equation (1) for As quantified in NGT, OGT, SNT, and STO canned foods marketed by the four Brazilian companies (G, C, O, and P).

In the CDI, we considered life exposure, ED = 8, 18, 30 years, and the ingestion of canned tuna of 130 g/day. The carcinogenic risk values were as follows: NGT = 1.8 × 10^−3^–6.2 × 10^−3^, OGT = 1.3 × 10^−3^–5.7 × 10^−3^, SNT = 1.2 × 10^−3^–5.4 × 10^−3^ and STO = 2.0 × 10^−3^–6.0 × 10^−3^ for As. The acceptable values are from 10^−6^–10^−4^.

Hazard quotients and hazard index (HI) for Al, As, Ba, Cu, Fe, Se, and Zn for canned tuna fish consumption for males, females, and children are shown in Table 3.

In Table 3, the hazard quotients for Se in canned tuna fish NGT-C and Ba in SNT-C are above one for 8-year-olds. All four brands’ hazard quotients (HQ) for arsenic surpass 1 for all studied population groups. When HQ > 1, there is a toxic risk to be considered. 

According to the calculated non-carcinogenic hazard index (HI), which is the sum of the risk quotients for simultaneous exposure to metals, that is, HI = HQ_Al_ + HQ_As_ + HQ_Ba_ + HQ_Cu_ + HQ_Fe_ + HQ_Se_ + HQ_Zn_ in each sample, there is a HI value superior to 1 for all studied population groups.

**Figure 2:**
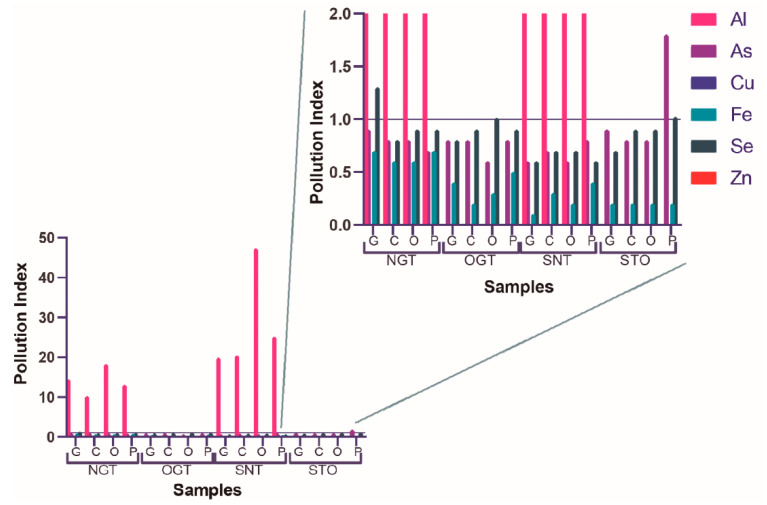
Pollution Index (PI) of the trace elements quantified in tuna fish.

The pollution index (PI) of the trace elements calculated in this study is presented in Figure 2.

Figure 2 shows that aluminum is the principal pollutant element, most quantified in canned tuna fish, with a PI value reaching 47.

Other elements such as selenium and arsenic presented PI > 1 for a few samples (Figure 2). Six samples—OGT-G, OGT-C, OGT-P, STO-G, STO-C, STO-O—were not contaminated by chemical elements.

## 4. Discussion

### 4.1. Canned Tuna Content and Intake Limits

The Al concentrations (Table 1) are higher than the content reported for canned tuna fish in Lebanon (0.62 mg/130 g) [13] and Turkey (0.70 mg/130 g) [56].

The aluminum consumption ranges from 21–69 mg/week for children (30 kg) and 14–105 mg/week for adults (70 kg) [57]. Aluminum exposure from foods can pose a higher risk to children, considering their body weight and the threat of achieving the threshold set by WHO of 2 mg/kg/week [53].

Aluminum is not an essential element for life, and is commonly considered toxic to humans; however, its toxicity depends on the route of exposure and solubility. This element accumulates in various body parts like tissues, such as the brain, bones, kidneys, and liver. Long-term exposure to low Al levels leads to toxic effects (Klotz et al., 2017 [26]), as well as prolonged exposure to low levels of aluminum leading to changes associated with brain aging and neurodegeneration [58]. In fact, the risk of consuming food with a high amount of Al is associated with Alzheimer’s diseases, Parkinson’s disease [27], bone disorder (competing with calcium and phosphate), kidney dysfunctions, anemia, gut dysfunctions, cytotoxic and neurotoxic, and others [57,59].

The arsenic (As) concentrations found in our results indicate that the arsenic is close to the levels reported for canned tuna in Iran (0.18 mg/130 g) [18] and Galicia in Spain (0.14–0.3 mg/130 g) [60]. On the other hand, As values in Table 1 are lower than the tuna commercialized in São Paulo in Brazil (0.57–1.53 mg/130 g) [17]. The only element that did not differ among several samples was arsenic (*p* > 0.05), present in similar amounts.

Safe daily provisions determined by the UL for men, women, pregnant women, and children have not yet been established. However, there are no safe levels for arsenic intake once this value was withdrawn [53]; therefore, we used the previous determination of a weekly limit consumption of 0.015 mg/kg, according to other studies [60,61,62]. In this instance, the concentrations of arsenic in Table 1 are above the tolerable daily intake limit values for this element set by FAO/WHO for foods (0.0021 mg/kg/day, equivalent to 0.147 mg/day for 70 kg adults). According to the Nationwide Food Consumption Survey (NFCS) in the US, the estimated daily arsenic intake is 20 mg/day for 6-year-old children, 47 mg/day for 40–45-year-old men, and 37 mg/day for 40–45-year-old women. Seafood contributes to around 90% of arsenic consumption for children (2-year-old), while other essential food sources such as rice represent only about 4% of arsenic intake; thus, the values of arsenic in tuna (Table 1) are below the values set by NFCS for As in food [63]. Long-term exposure to arsenic from drinking water and food can cause cancer and bladder cancers [64]. In fact, the International Agency for Research on Cancer (IARC) has classified arsenic and arsenic compounds as carcinogenic to humans. This is based on sufficient evidence in humans that these compounds can cause: respiratory dysfunctions, gastrointestinal and neuro-cardiovascular diseases, anemia disorder, liver disorder, leucopenia, and thrombocytopenia, diabetes, cytotoxic, and genotoxicity effects [64,65,66].

Barium showed high concentrations only in samples of natural solid tuna in two brands (Figure 1). The Ba contents in OGT and STO are near the reported values for canned tuna fish (0.06 mg/130 g) purchased in Jordanian markets. The SNT samples had a higher barium content than those reported in this same study [12], as well as from samples from New Zealand and the United Kingdom (0.52–17.03 mg/130 g) [67].

So far, there is no UL set for barium. On the other hand, barium’s tolerable daily intake limit values are determined by FAO/WHO [38] 0.02 mg/kg/day in drinking water (equivalent to 1.4 mg/day for 70 kg adults). Besides, Montanari (2015) [68] described barium as a part of metal contaminants, where barium sulfate is used to manufacture cans and lids as an inorganic charge. Considering the threshold proposed by the Commission Regulation 10/2011 of 1 mg/kg for specific migration from packaging compounds to food [69], we can say that the values of Ba in NGT, STO, and OGT are below these limits, while SNT-C and SNT-O are above it.

Barium accumulation can occur from an occupational hazard or from the consumption of contaminated water and food. The average amount of ingestion worldwide and its geographic variation are unknown, due to a lack of research attention. The presence of the element can produce different effects, especially in cases of exposure, either in low or moderate doses. Information on the potential health effects of barium exposure is primarily from animal studies and reported to encompass renal, neurological, cardiovascular, mental, and metabolic diseases [31].

The quantified calcium levels in SNT and STO are near the concentrations reported for raw and steamed tuna fish (4%) in Spain [70], while NGT and OGT presented a lower calcium concentration. Structure and solvent were the main sources (95.79%; *p* < 0.0001) of calcium variations in samples, with high calcium content in solid samples only.

Calcium is an essential element, and its adequate consumption is related to a minor risk of hypertensive disorders, lower blood pressure, lower cholesterol values, osteoporosis reduction, bone resorption, and others, while in a higher concentration, it is associated with renal stones formation and myocardial infarction in older humans [70].

The permissible limit of Ca set by UL is 2500 mg/day. Comparison between detected values and UL set value considers calcium concentration in the samples below the proposed limits, not posing a risk from this element intake [36].

As for copper concentrations in the samples, the OGT-P sample showed the highest concentration with an average copper concentration of 0.10 ± 0.01 mg/130 g; these values are close to the tuna obtained in Egypt (0.16 mg/130 g) [71] and higher than those found in Turkey (0.0026 mg/130 g) [20]. Solvent and structure were critical to copper variation in samples, accounting for 61.4% (*p* < 0.0001), followed by the interaction with the brands (28.74%; *p* = 0.0001); grated samples and samples in oil registered higher copper amounts. The brand alone was responsible for only 3.88% of variations (*p* = 0.04).

There is no established UL for Cu at the moment. However, all Cu concentrations in canned tuna are below the tolerable daily intake limit value set by FAO/WHO for Cu (0.5 mg/kg/day, equivalent to 35 mg/day for 70 kg adults). Therefore, the ingestion of these canned tuna samples should be safe for consumption regarding copper content. Adequate copper consumption promotes health benefits, correlating with good functionality of the cardiovascular system, lower blood glucose, cholesterol, and lipid levels [72], cognitive, and is not associated with arthritis or cancer, cofactor, antioxidant effects, oxidative activity, absorption, and others [73]; whereas, elevated copper intake is related to mitochondrial dysfunction [74], liver damage, and Alzheimer’s disease [75]. Disturbances in the copper metabolism due to genetic conditions can result in copper deficiency (Menkes syndrome) and toxicity (Wilson’s disease) [76].

The iron content observed in this study agrees with the reported systematic review studies of canned tuna fish (1.71 mg/130 g) in Iran [77], and it was consistent with Fe content found in Mediterranean wild Atlantic Bluefin tuna (about 1.7 mg/130 g) [78]. The two-way ANOVA identified that variations in iron were due to structure and solvent (81.94%; *p* < 0.0001), brand (8.045%; *p* < 0.0001), and the interaction between these factors (7.525%, *p* = 0.018).

The UL for males, females, and pregnancy is 45 mg/day of iron, while for children, it is 40 mg/day. In addition, the tolerable daily intake limit value established by the FAO/WHO [34] is 0.8 mg/kg/day, equivalent to 56 mg/day for 70 kg adults. Thus, the concentration of iron in canned tuna samples is unlikely to cause adverse health effects. Iron levels adequacy is correlated with maximal oxygen respiration and exercise performance, electron transport, hemoglobin synthesis, immunity, anemia prevention, pregnancy development, deoxyribonucleic acid synthesis, gut microbiota health modulation, neurodevelopment, and others [79,80].

The Se contents observed in our study are according to those reported (0.0169–0.5850 mg/130 g) for canned fish marked in Iran [22], and it is consistent with Se content found in Atlantic Bluefin tuna from the Mediterranean Sea (about 0.143 mg/130 g) [81]. The variations in selenium amount mainly depend on the structure and solvent (53.01%; *p* < 0.0001), while brand represents just 8.064% of the difference, and the interaction between factors is 37.17%.

Selenium detections are below the UL for the consumption of Se in male/female (0.4 mg/day) and children (0.15 mg/day). However, these values are higher than the value established by FAO/WHO for pregnant women (0.06 mg/day) [82]. Food toxicity of selenium in humans is rare; however, selenium (IV) is generally more toxic than selenium (VI). Excessive selenium intake can lead to selenosis, dermatitis, alopecia, elevated mortality rate, an enhanced risk for prostate cancer, and non-melanoma skin cancer [83]. On the other hand, sufficient selenium intake is associated with preventing and decreasing diabetes mellitus, cancers [22,84], improving male fertility, human neuropathies, and hepatic steatosis [85,86,87].

The concentration of Zn in all samples in Table 1 is below the study reported on the levels of selected heavy metals in canned tuna fish produced in Turkey for Zn (1.066–1.482 mg/130 g for Zn) [20]. Zinc quantities varied according to structure and solvent (52.04%, *p* < 0.0001), followed by the interaction between factors (33.70%, *p* < 0.0001) and brand (13.43%, *p* < 0.0001).

The values of Zn set by UL for male/female and pregnant woman are 40 mg/day, and children 5 mg/day, while the values established by FAO/WHO [35] are 1 mg/kg/day, equivalent to 70 mg/day for 70 kg adults. All zinc values in samples are below the values set by UL and FAO/WHO. Thus, they should be safe for human consumption regarding this element. Zinc has a critical effect on homeostasis; immune function in oxidative stress. However, high doses of this element have toxic effects, making acute zinc intoxication a rare event [88].

Regarding the content of non-essential elements (Figure 1), a great difference was noted between the aluminum contents in samples preserved in brine (NGT and SNT) and in oil. In the natural samples (brine–NGT and SNT), aluminum detection presented a substantial amount opposite to those in oil. Aluminum can migrate from the cans to the food, and some conditions may facilitate this transference. Our results agree with the Stahl et al. (2017) findings [59], where aluminum migrated in a more critical matter in acidic water, water, and then oils.

Another significant point is the presence of arsenic in all samples, which is toxic and non-essential. The previous limit of consumption was withdrawn once they could no longer be considered safe [37]. Therefore, while the excess of some elements can pose a hazard for human intake, the mere presence of others can already raise a red flag.

In the following subsection, we will discuss the potential risks of the elemental content in canned samples. The potential risks of arsenic content in canned tuna were verified using the carcinogenic risk equation.

### 4.2. Health Risk Assessment

In the calculation of CDI, we considered life exposure, ED = 8, 18, 30 years, and ingestion of canned tuna of 130 g/day. The carcinogenic risk (CR) values obtained for 8-year-old children were higher in the NGT-G sample when compared to other companies (C, O, and P) and types of canned tuna fish (OGT, SNT, and STO). The carcinogenic risk values for NGT (1.8 × 10^−3^–6.2 × 10^−3^), OGT (1.3 × 10^−3^–5.7 × 10^−3^), SNT (1.2 × 10^−3^–5.4 × 10^−3^), and STO (2 × 10^−3^–6 × 10^−3^) for As are higher than the acceptable values (10^−6^–10^−4^); that is, all CR for arsenic are considerable unacceptable in these canned tuna samples. Arsenic is the main contaminant trace element that can be correlated with several cancer incidences among all heavy metals in the canned tuna fish samples. Furthermore, the total cancer risk incidence can increase for those who consume the recommended 150 g/day [11] of canned tuna fish of types NGT, OGT, SNT, and STO, with an elevated risk for the youngest ones.

The risk assessment can provide information on non-cancerous health risks through the HQ factors (hazard quotient). The risk quotient for Se in NGT-C canned tuna and Ba in SNT-C (Table 3) is greater than one for 8-year-olds. The risk quotients (HQ) of all four brands for arsenic exceed 1 for all groups studied.

When HQ > 1, there is a toxic risk to be considered. All HQ values for Al, Fe, Cu, and Zn for all studied populations are below 1, indicating no potential health risk through canned tuna consumption for each element [51].

According to the calculated non-carcinogenic hazard index (HI), which is the sum of the risk quotients for simultaneous exposure to metals, that is, HI = HQ_Al_ + HQ_As_ + HQ_Ba_ +HQ_Cu_ + HQ_Fe_ + HQ_Se_ + HQ_Zn_ in each sample, there is a HI value superior to 1 for all studied population groups, indicating that canned tuna consumption from local markets can pose a risk for human health regarding metal and metalloid content. The HI in the Table 3 was higher for children than adults. The HI values in Table 3 are higher than those obtained in a study carried out in China with marine fish, with HI = 0.945 for adults. However, the results for children (HI = 8.556) published by Han et al. (2021) are within the values obtained for children in Table 3 [89]. Arsenic was the element that contributed the most to an elevated HI in most canned tuna samples (Table 3) and marine fish [89].

In general, the presence of the chemical elements Al, As, Se, Cu, Fe, and Ba in all types of canned fish can be explained by a higher occurrence of these metals and metalloids in various kinds of water springs, lakes, streams, rivers, seas, and oceans, mainly linked to anthropogenic activities [2,3,4]. In addition, Fe and Al in canned tuna samples may be explained by the migration of these elements from the can package into the fish [13]. Besides, tuna was recognized as a predator able to concentrate large amounts of heavy metals [90]. Therefore, with the consumption of 130 g/day of canned tuna fish, these chemical elements can be a non-carcinogenic hazard to human health.

The concentration of metals in fish relates to environmental pollution; since fish can accumulate pollutants from the surrounding area [91]. While fatty tissues like the liver accumulate in most metals, muscle tissue has lower contents [92,93].

The pollution index values of the samples are represented in Figure 2. For the interpretation of the pollution index results, it is known that a PI above 1 indicates sample contamination, and can be considered toxic [47]. Aluminum is the main pollutant, the most quantified in canned tuna, with a PI value of 47.

According to Hydes, besides anthropogenic factors, those responsible for the high concentration of aluminum in the sea are the clayey sediments that probably arise from biological activities [94], originating from the aluminum of atmospheric particles and by the balance in the sediment through the silicon generated by the death of aquatic organisms [95]. On the other hand, a high amount of Al in canned fish may be correlated with the interaction between aluminum foil particles in sauced food, which is potentially hazardous to several metals, skeleton diseases, cancers, and so on [58]. The use of internal coatings reduced the metal migration to the food (Al, Fe, Cd, Sn, and Pb) [96], but it still happens, mainly due to the discontinuous or not compact coating of the materials [97].

Other elements such as selenium and arsenic presented PI > 1 for a few samples (Figure 2). Sediments can be a significant source of selenium in fish and invertebrates. Toxic effect threshold levels for selenium in fish have been reported as 4 mg/kg (for whole fish) [98]. Therefore, high content calculated to Se in canned tuna fish can be explained by bioaccumulation in its several tissues from the water of environment, plankton, and other food types in the chain consumption [99]. In addition to anthropogenic factors, marine algae release arsenite into the seawater, which is toxic to marine phytoplankton, marine invertebrates, and fish. Tissues of marine invertebrates and fish contain high concentrations of arsenic. Therefore, marine arsenic represents a low risk to human consumers of fishery products [100]. The samples that did not indicate contamination by chemical elements were: OGT-G, OGT-C, OGT-P, STO-G, STO-C, STO-O.

Tuna fish and other fish species are critical in the human diet and represent a source of protein, but they may accumulate potentially toxic metals. According to the First World Ocean Assessment released in 2015, many ocean parts had been seriously degraded. The results obtained with Brazilian tuna, tuna marketed in Iran, Egypt, Turkey, Thailand, and other studies indirectly show that ocean degradation still remains and has increased over the years, affecting some fish species. As an alternative, the United Nations has proclaimed the Decade of Ocean Science for Sustainable Development (2021–2030) to support efforts to reverse the cycle of decline in ocean health [101].

## 5. Conclusions

All the canned tuna fish samples that we studied accumulated heavy metals. The majority of elements (Al, Fe, Ba, Ca, Cu, and Zn) in Brazilian canned tuna fish complied with the permissible limits by UL and FAO/WHO, while As in all samples, and Ba in SNT, is above these thresholds. The Se levels in our canned tuna fish are elevated for pregnant women consumption by the limit established by FAO/WHO.

The carcinogenic risk (CR) values due to the ingestion of Brazilian canned tuna fish obtained for 8-year-old children are higher than for adolescents and adults, related to the expected weight. The carcinogenic risk values for As are above acceptable values set by US EPA (≥10^−4^) in all samples, and a potential hazard.

The primary contaminant in the samples was aluminum, in large amounts in samples in brine. While aluminum presented higher quantities, despite lower arsenic concentrations, they proved unacceptable, contributing to the general Hazard Index in all samples.

Considering the pollution index, Al, Se, and As are the principal pollutants, with samples achieving PI values above 1. Since the canned tuna samples had a high concentration of heavy metals, they could serve as bioindicators of seas and oceans pollution that may be contaminated with various heavy metals. Yet, it is not possible to disregard the role of packaging contaminations for some elements, such as aluminum.

With the high concentration of heavy metals in our samples and other studies, it is not safe to maintain the annual intake established by the FAO/FDA for fish consumption when canned samples are the only source, considering that the average serving amount of canned tuna sold in Brazil is 130 g, which can be harmful from an elementary point of view. Lower amounts may be more adequate. This statement does not mean that fresh fish is free from contamination, and further studies should be carried out.

## Figures and Tables

**Table 1:** Concentrations of metals and metalloids in canned tuna (NGT, OGT, SNT, and STO) of the company’s four brands (G, C, O, and P) compared with FAO/WHO limits.

Element	Natural Grated Tuna—NGT (mg/130 g)				Oil Grated Tuna—OGT (mg/130 g)				Solid Natural Tuna—SNT (mg/130 g)				Solid Oil Tuna—STO (mg/130 g)				Reference for 70 kg Adults (mg/day)
	NGT-G	NGT-C	NGT-O	NGT-P	OGT-G	OGT-C	OGT-O	OGT-P	SNT-G	SNT-C	SNT-O	SNT-P	STO-G	STO-C	STO-O	STO-P	
Al	1.9 ± 0.4	1.3 ± 0.003	2.4 ± 0.4	1.7 ± 0.3	<LOD	<LOD	<LOD	<LOD	2.6 ± 0.03	2.7 ± 0.4	6.1 ± 0.4	3.3 ± 0.5	<LOD	<LOD	<LOD	0.00065 ± 1.3 × 10^−5^	19.95 [37]
As	0.3 ± 0.01	0.2 ± 0.007	0.2 ± 0.004	0.2 ± 0.01	0.2 ± 0.001	0.2 ± 0.01	0.2 ± 0.006	0.2 ± 0.03	0.2 ± 0.002	0.2 ± 0.01	0.2 ± 0.003	0.2 ± 0.006	0.2 ± 0.006	0.2 ± 0.001	0.2 ± 0.01	0.2 ± 0.02	0.147 [39]
Ba	<LOD	<LOD	<LOD	<LOD	<LOD	<LOD	0.004 ± 0.02	0.002 ± 0.0003	<LOD	15 ± 4	10 ± 4.9	<LOD	0.008 ± 0.003	0.03 ± 0.007	0.08 ± 0.03	0.02 ± 0.004	1.4 [38]
Ca	0.07 ± 0.03	0.01 ± 0.003	0.03 ± 0.003	0.03 ± 0.006	<LOD	<LOD	<LOD	0.04 ± 0.005	5.4 ± 0.2	5.2 ± 0.03	5.8 ± 0.1	6.2 ± 0.04	9 ± 0.2	8.2 ± 0.4	9.2 ± 2.6	8.9 ± 0.4	2000–2500 [36]
Cu	0.08 ± 0.004	0.07 ± 0.03	0.09 ± 0.01	0.04 ± 0.01	0.06 ± 0.02	0.03 ± 0.005	0.04 ± 0.006	0.1 ± 0.013	<LOD	0.008 ± 0.002	<LOD	0.02 ± 0.004	0.04 ± 0.004	0.02 ± 0.003	0.03 ± 0.005	0.03 ± 0.004	3 [35]
Fe	3.9 ± 0.5	3.3 ± 0.03	3.3 ± 0.06	4 ± 0.2	2.3 ± 0.6	1.4 ± 0.1	1.8 ± 0.7	2.7 ± 0.4	1 ± 0.059	2 ± 0.04	1 ± 0.06	2.2 ± 0.2	1.2 ± 0.02	1.1 ± 0.04	1 ± 0.009	1.4 ± 0.106	17 [34]
Se	0.3 ± 0.02	0.2 ± 0.003	0.3 ± 0.005	0.2 ± 0.01	0.2 ± 0.01	0.3 ± 0.01	0.3 ± 0.05	0.3 ± 0.03	0.2 ± 0.001	0.2 ± 0.01	0.2 ± 0.001	0.2 ± 0.005	0.2 ± 0.01	0.2 ± 0.005	0.26 ± 0.01	0.3 ± 0.02	0.04 [36]
Zn	0.05 ± 0.005	0.02 ± 0.0003	0.04 ± 0.001	0.03 ± 0.001	0.03 ± 0.001	0.01 ± 0.001	0.03 ± 0.004	0.05 ± 0.006	0.01 ± 4 × 10^−6^	0.02 ± 0.001	0.01 ± 0.0006	0.02 ± 0.001	0.02 ± 0.0002	0.01 ± 0.001	0.02 ± 0.002	0.013 ± 0.001	70 [35]

Note: <LOD—analyte concentration below the limit of detection; ND = not determined.

**Table 2:** Life exposure (Age = 8, 18, 30 years), values of carcinogenic risk (CR) due to exposure of As in canned NGT, OGT, SNT, and STO from the four companies (G, C, O, and P), considering a daily intake of 130 g/day.

Sample	Cancer Risk Arsenic (As)		
	8 Years Old	18 Years Old	30 Years Old
**NGT-G**	0.0062	0.0026	0.0023
**NGT-C**	0.0051	0.0022	0.0019
**NGT-O**	0.0053	0.0022	0.0020
**NGT-P**	0.0047	0.0020	0.0018
**OGT-G**	0.0057	0.0024	0.0013
**OGT-C**	0.0054	0.0023	0.0020
**OGT-O**	0.0044	0.0019	0.0016
**OGT-P**	0.0052	0.0022	0.0019
**SNT-G**	0.0043	0.0018	0.0016
**SNT-C**	0.0049	0.0021	0.0018
**SNT-O**	0.0041	0.0017	0.0015
**SNT-P**	0.0054	0.0022	0.0012
**STO-G**	0.0060	0.0025	0.0022
**STO-C**	0.0054	0.0023	0.0020
**STO-O**	0.0056	0.0024	0.0021
**STO-P**	0.0060	0.0025	0.0022

**Table 3:** Hazard quotient (HQ) and Hazard index (HI) due to the ingestion of canned tuna fish commercialized in Brazil for individuals aged 8, 18, and 30 years old.

Age (Years)	Samples	HQ							HI	Samples	HQ							HI
		Al	As	Ba	Cu	Fe	Se	Zn			Al	As	Ba	Cu	Fe	Se	Zn	
**8**	NGT-G	0.03	13.84	0.00	0.03	0.09	1.11	0.003	15.11	SNT-G	0.04	9.47	0.00	0.00	0.02	0.54	0.0007	10.08
	NGT-C	0.02	11.48	0.00	0.03	0.08	0.74	0.001	12.35	SNT-C	0.04	10.96	1.25	0.003	0.05	0.62	0.001	11.91
	NGT-O	0.04	11.70	0.00	0.04	0.08	0.85	0.002	12.75	SNT-O	0.10	9.10	0.80	0.00	0.03	0.63	0.0006	10.64
	NGT-P	0.03	10.50	0.00	0.02	0.09	0.81	0.002	11.44	SNT-P	0.05	11.90	0.00	0.007	0.05	0.54	0.0009	12.54
	OGT-G	0.00	12.70	0.00	0.02	0.05	0.74	0.002	13.52	STO-G	0.00	13.36	0.0006	0.018	0.03	0.66	0.001	14.05
	OGT-C	0.00	12.06	0.00	0.01	0.03	0.83	0.0008	12.93	STO-C	0.00	11.99	0.003	0.01	0.03	0.85	0.0008	12.88
	OGT-O	0.00	9.82	0.0003	0.02	0.04	0.87	0.001	10.75	STO-O	0.00	12.51	0.001	0.01	0.03	0.85	0.0009	13.40
	OGT-P	0.00	11.47	0.0001	0.04	0.07	0.84	0.003	12.41	STO-P	0.00001	13.23	0.001	0.01	0.03	0.88	0.0007	14.19
**18**	NGT-G	0.01	5.80	0.00	0.01	0.04	0.47	0.001	6.33	SNT-G	0.02	3.97	0.00	0.00	0.01	0.23	0.0003	4.23
	NGT-C	0.01	4.82	0.00	0.01	0.03	0.31	0.0005	5.18	SNT-C	0.02	4.60	0.52	0.001	0.02	0.26	0.0005	5.41
	NGT-O	0.02	4.90	0.00	0.01	0.03	0.36	0.0008	5.30	SNT-O	0.04	3.82	0.33	0.00	0.01	0.26	0.0003	4.46
	NGT-P	0.01	4.40	0.00	0.006	0.04	0.34	0.0007	4.80	SNT-P	0.02	4.99	0.00	0.003	0.021	0.23	0.0004	5.26
	OGT-G	0.00	5.33	0.00	0.01	0.02	0.31	0.0007	5.67	STO-G	0.00	5.60	0.0003	0.008	0.012	0.28	0.0005	5.90
	OGT-C	0.00	5.06	0.00	0.01	0.01	0.35	0.0003	5.42	STO-C	0.00	5.03	0.0010	0.004	0.01	0.36	0.0003	5.40
	OGT-O	0.00	4.12	0.0001	0.01	0.02	0.37	0.0006	4.50	STO-O	0.00	5.25	0.0006	0.004	0.01	0.36	0.0004	5.60
	OGT-P	0.00	4.81	0.00006	0.02	0.027	0.35	0.001	5.21	STO-P	0.000004	5.57	0.0006	0.0047	0.0140	0.37	0.0003	5.95
**30**	NGT-G	0.01	5.14	0.00	0.01	0.03	0.41	0.0011	5.61	SNT-G	0.01	3.51	0.00	0.00	0.01	0.20	0.0003	3.74
	NGT-C	0.01	4.27	0.00	0.01	0.03	0.27	0.0004	4.58	SNT-C	0.02	4.07	0.46	0.0013	0.02	0.23	0.0004	4.80
	NGT-O	0.01	4.34	0.00	0.01	0.03	0.31	0.0007	4.71	SNT-O	0.04	3.38	0.29	0.00	0.01	0.23	0.0002	3.96
	NGT-P	0.01	3.90	0.00	0.01	0.03	0.30	0.0007	4.24	SNT-P	0.01	2.65	0.00	0.002	0.01	0.12	0.0002	2.79
	OGT-G	0.00	2.83	0.00	0.01	0.01	0.17	0.0004	3.01	STO-G	0.00	4.96	0.0002	0.007	0.01	0.24	0.0004	5.22
	OGT-C	0.00	4.48	0.00	0.004	0.01	0.31	0.0003	4.80	STO-C	0.00	4.45	0.0009	0.003	0.01	0.32	0.0003	4.78
	OGT-O	0.00	3.65	0.00012	0.006	0.02	0.32	0.0005	3.99	STO-O	0.00	4.65	0.0005	0.004	0.01	0.32	0.0003	4.98
	OGT-P	0.00	4.26	0.00005	0.01	0.02	0.31	0.001	4.61	STO-P	0.000004	4.93	0.0005	0.004	0.01	0.32	0.0003	4.65

## Data Availability

Data will be available upon reasonable request to the corresponding author.
